# Intra-articular tracking of adipose-derived stem cells by chitosan-conjugated iron oxide nanoparticles in a rat osteoarthritis model†

**DOI:** 10.1039/c8ra09570a

**Published:** 2019-04-16

**Authors:** Meihua Xie, ShuLin Luo, Ying Li, Laiya Lu, Cuijun Deng, Yu Cheng, Feng Yin

**Affiliations:** Institute for Regenerative Medicine, The Institute for Biomedical Engineering & Nano Science, Shanghai East Hospital, Tongji University School of Medicine 1800 Yuntai Road Shanghai 200123 China yucheng@tongji.edu.cn; Department of Joint Surgery, Shanghai East Hospital, School of Medicine, Tongji University Shanghai China 001yinfeng@sina.com

## Abstract

Adipose-derived stem cells (ADSCs) hold great potential in cartilage tissue engineering due to their multipotency and ease of availability. MRI is an effective and noninvasive imaging approach to track cells and observe new tissue regeneration. It is essential to find a compatible and efficient imaging reagent without affecting the stemness of ADSCs. Herein, we developed chitosan-modified iron oxide nanoparticles (IO-CS) as the *T*_2_ contrast reagent with good cell compatibility and high cellular uptake efficiency and used IO-CS for ADSC intra-articular imaging in a rat osteoarthritis (OA) model. TEM demonstrated the great morphology and size distribution of IO-CS nanoparticles with the size of 17 nm. Magnetization (29.4 emu per g) and MRI tests confirmed (*R*_2_ of 184 mM^−1^ s^−1^) the feasibility of IO-CS nanoparticles as an MRI contrast reagent. In addition, the IO-CS nanoparticles showed good cellular compatibility and high labeling efficiency as compared to the commercial agent ferumoxytol. Moreover, incorporation of IO-CS nanoparticles did not alter the adipogenic, osteogenic and chondrogenic differentiation ability of ADSCs. Furthermore, the MRI transverse *R*_2_ maps showed a persistence time of the IO-CS nanoparticles in ADSCs of 6 days *in vitro*. Then, we investigated the imaging capability of the IO-CS nanoparticle-labeled ADSCs *in vivo* with MRI for 5 weeks. The histological studies demonstrated the intra-articular biodistribution of the IO-CS nanoparticles, including in the cartilage superficial layer, synovial sublining layer, periosteum and bone marrow cavity. They provided systemic distribution information of the ADSCs in the OA rat model. In summary, we developed an accessible and effective *T*_2_ imaging reagent with good biocompatibility and maintenance of the stemness of ADSCs. This showed the potential translational application of IO-CS nanoparticles as an MRI reagent in cartilage tissue engineering.

## Abstract 2

The chitosan modified IO-CS nanoparticles are biocompatible and efficient MRI reagents for the adipose-derived stem cell labeling and tracking in an osteoarthritis rat model.

## Graphical abstract

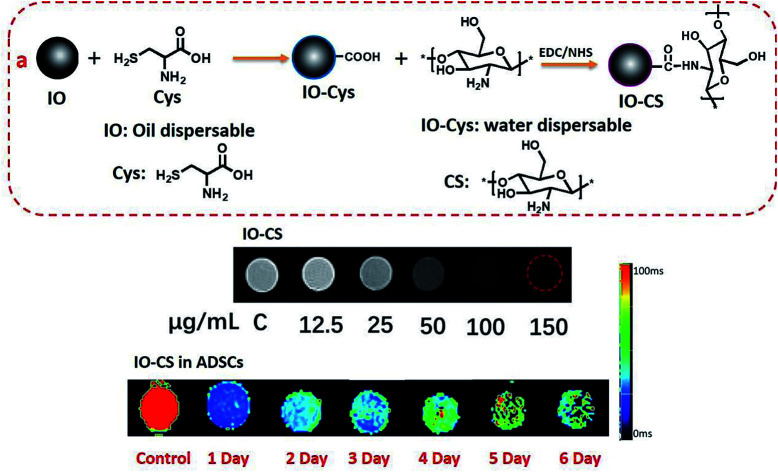

## 1. Introduction

Osteoarthritis (OA) is the most common degenerative joint disease and still far from cure. Stem cell therapy is widely applied in tissue regeneration,^1,2^ cancer treatment^3,4^ and wound healing.^5^ This stimulates researchers to restore diseased articular cartilage in OA patients *via* stem cell therapy.^1,6^ Although some studies have demonstrated the effectiveness and potential of stem cell therapy in OA cartilage regeneration, to date, there is a lack of tools for tracking the stem cells. Cell imaging reagents, including organic materials (fluorescent small molecules,^7^ fluorescent proteins,^8^ carbon dots^9^) and inorganic materials (quantum dots,^10^ gold nanoparticles,^11^ silicon nanoparticles^12^ and iron nanoparticles^13^) have been developed to visualize the localization of stem cells. For live cell imaging, whether *in vitro* or *in vivo*, the imaging agents should label the cells without affecting the function of the cells. Specially, cartilage regeneration involves a long-lasting process accompanied by cell proliferation and differentiation. Usually, the periods of observation ranged from 5 weeks to 10 weeks, and clinical trials could last for years or even longer.^14–16^ Still, it is urgent to develop a safe, long-term and effective imaging reagent for stem cells in the treatment of OA cartilage.

Magnetic resonance imaging (MRI) is a powerful and noninvasive tracking method, and iron oxide (IO) nanoparticles are popular contrast agents for MRI.^17,18^ IO nanoparticles are preferred tools in biomedical engineering for therapeutics, magnetic separation technologies and diagnostics.^19,20^ MRI instruments associated with IO nanoparticles could be an effective tracking method with excellent spatial resolution at any depth by labeling stem cells.^17^ In addition, IO nanoparticles can be facilely fabricated by physical, chemical and biological approaches.^19^ Most importantly, it was confirmed that IO nanoparticles were safe and effective in tracking human chondrocyte,^21^ human bone marrow-derived mesenchymal stem cells,^22^ and neural stem cells.^23^ Ferumoxytol is an FDA-approved commercial iron supplement and has been used as an MRI reagent for clinical trials to track stem cells. However, the low uptake of ferumoxytol is still a challenge for cell labeling and tracking *via* MRI.^24,25^ It is meaningful to develop a feasible MRI platform with compatibility and efficiency for pre-clinical and clinical studies.

Scientists had developed a surface coating methodology to improve the biocompatibility and cellular uptake of IO nanoparticles. Some reports have shown modified iron oxide nanoparticles serving as cell imaging and drug delivery vectors for bone marrow-derived mesenchymal stem cells,^22,26,27^ neural stem cells,^23^ immune cells,^18^ chondrocytes,^21^ and adipose-derived stem cells (ADSCs),^28^ most of which showed low uptake and low labelling efficiency. There were several polymers for surface coating such as dextran, starch and polyol derivatives. Chitosan is a positively charged natural polymer and was used to modified IO to track stem cells in rabbit ischemic brains for 16 days.^26^ This could limit the imaging duration because labeled IO content would dilute upon cell division and metabolism. For OA cartilage regeneration, cells should be accurately detected for long-term tracking for clinical trials. ADSCs are pluripotent cells, which could form cartilage, bone and adipose.^2,29–31^ ADSCs have demonstrated efficacy in the preclinical and clinical treatment of OA *via* intra-articular injection, and the easy access of ADSCs makes them potentially beneficial in the regeneration of cartilage tissue.^29^ We aimed to fabricate a feasible and efficient MRI contrast agent to investigate the persistence of ADSCs for preclinical and clinical trials.

In this work, we presented chitosan-modified iron oxide (IO-CS) nanoparticles for ADSC imaging with the long MRI effect *in vitro* and *in vivo*. IO-CS nanoparticles were easily obtained with a facile process in the aqueous solution. TEM and DLS measurements demonstrated the great morphology and size distribution of IO-CS nanoparticles. Magnetization (29.4 emu per g) and MRI tests confirmed (*R*_2_ of 184 mM^−1^ s^−1^) the feasibility of IO-CS nanoparticles as an MRI reagent, and IO-CS nanoparticles showed good cell compatibility of up to 200 μg Fe per mL concentration and high cellular uptake effect. High cellular uptake enhanced cell tracking effect and prolonged the imaging time. ADSCs labeled with IO-CS nanoparticles showed multipotential differentiation, including adipogenic, osteogenic and chondrogenic differentiation capacity. Furthermore, IO-CS nanoparticles could track ADSCs *in vitro* and *in vivo* for 1 week and 5 weeks, respectively. IO-CS nanoparticles demonstrated a stable and good imaging effect in ADSCs in the OA animal model. Additionally, the intra-articular biodistribution of IO-CS nanoparticles covered the cartilage superficial layer, synovial sublining layer, periosteum and bone marrow cavity. This study reported a practical tool for tracking stem cells after intra-articular injection, which was an effective and stable tracer of ADSCs in the OA rat model. IO-CS nanoparticles are a good candidate as an MRI reagent for clinical imaging.

## 2. Experimental

### 2.1 Materials

FeCl_3_·6H_2_O and cysteine were purchased from Macklin (Shanghai, China). 1-Octadecene was purchased from TCI Co., Ltd (Shanghai, China). Ferumoxytol (Feraheme injection, AMAG Pharmaceuticals, MA) was used according to the instructions after purchase. Sodium oleate, ethyl alcohol, hexane, isopropanol and methylbenzene were purchased from Sinopharm Chemical Reagent Co., Ltd (Shanghai, China). Chitosan was purchased from Aladdin (Shanghai, China). Phosphate buffer saline (PBS) and low glucose Dulbecco's modified Eagle's medium (DMEM) were purchased from HyClone (America). Penicillin/streptomycin and fetal bovine serum (FBS) were purchased from Gibco (America). Basic fibroblast growth factor (bFGF) was purchased from Invitrogen (America). The Cell Counting Kit-8 (CCK8) was purchased from Dojindo Co., Ltd (Japan).

### 2.2 Synthesis of iron oxide nanoparticles

Iron oxide nanoparticles were synthesized according to the report by Gao *et al.*^32^ with some modifications. Briefly, 4.1 g (15.17 mmol) of FeCl_3_·6H_2_O and 13.7 g (45.00 mmol) of sodium oleate were dissolved in a mixture of 40 mL of ethyl alcohol, 60 mL of double distilled (DD) water and 40 mL of hexane. The resulting solution was heated to 70 °C and kept for 4 hours. Then, the product was washed three times with DD water. 3.6 g of the obtained iron oleate (3 mmol) was dispersed in 20 mL of 1-octadecene and 1.6 mL of oleic acid. The reactants were purified with argon and kept at 120 °C for 30 min. Then, the mixture was heated to 320 °C and maintained for half an hour. The resulting black product was washed three times with methylbenzene and dried in a vacuum.

### 2.3 Synthesis of chitosan modified iron oxide (IO-CS)

Next, iron oxide (IO) nanoparticles were modified with chitosan. Firstly, the oil-dispersible iron oxide nanoparticles were turned into water-dispersible nanoparticles by attaching cysteine on the surface of IO. The specific steps were as follows: 10 mg of iron oxide powder was added to 10 mL of anhydrous dimethylformamide and 50 mg of cysteine dissolved in 2 mL of water was added into the above solution. Then, the mixture was stirred for 48 h at room temperature. The cysteine-modified iron oxide nanoparticles (IO-Cys) were obtained and washed three times with alcohol and water, respectively. To obtain chitosan-modified iron oxide nanoparticles, the obtained IO-Cys were dissolved in 10 mL of DD water containing 76 mg of EDC and 69 mg of NHS and was then activated for 45 min. The activated IO-Cys were separated by magnetic separation and then re-dispersed in 5 mL of water. 5 mL of 1 wt% chitosan with 0.5% acetic acid was added dropwise to the IO-Cys solution with vigorously stirring for the next 24 h at room temperature. The product was obtained by magnetic separation and stored in DD water.

### 2.4 Characterizations

The morphology of the IO and IO-CS nanoparticles were observed using an FEI Tecnai F30 scanning transmission electron microscope (TEM) with a Gatan CCD digital micrograph. The hydrodynamic size of the IO-CS nanoparticles was measured by dynamic light scattering (DLS). Zeta potential measurements were performed on a Malvern Zetasizer Nano ZS apparatus. The magnetism of the magnetic nanoparticles was investigated with a 0.5 T Physical Property Measurement System (PPMS-9, Quantum Design, America) at 300 K. A CCK-8 assay was carried out to measure the stem cell viability according to the instructions, and then, the absorption of the medium was read on a microplate reader (ELx808, BioTek) at 450 nm. The transverse plane MRI was scanned on a Germany 3 T MRI scanner (MAGNETOM Prisma, Siemens Healthcare, Erlangen). All pictures of cells were shot using the Living Cell imaging inversion microscope (IX73, Olympus, China). Inductively coupled plasma mass spectrometry (ICP-MS, Thermo Fisher, iCAP Q) equipment was used to measure the iron content of IO and IO-CS.

### 2.5 Preparation of ADSCs

ADSCs were prepared according to the method developed by Jingjing Fan *et al.* with some modifications.^33^ In brief, adipose tissue was obtained from the inguinal regions of 4 week-old male Sprague-Dawley rats (SD, Vital River, Beijing, China). The adipose tissue was minced with surgical scissors and digested with collagenase I (2 mg mL^−1^; Worthington Biochemical Corp, Lakewood, NJ, USA) at 37 °C for 45 minutes. The digested mixture was filtered with a 70 μm membrane and then centrifuged at 2000 rpm for 10 min to eliminate the undigested fragments. The pellet at the bottom of the tube was resuspended in the stem cell culture medium (DMEM + 15% FBS) and cultivated for 72 hours at 37 °C in 5% CO_2_ atmosphere. Unattached cells and debris were removed, and fresh culture medium was added to the culture at 37 °C in 5% CO_2_. Passage 4 cells were used for the following experiments.

### 2.6 *In vitro* cellular labeling

IO-CS nanoparticles were sterilized in penicillin and streptomycin solution. They were washed three times with sterilized PBS and dispersed in the cell culture medium with ultrasound. The concentrations of IO-CS nanoparticles in the cell culture medium were 12.5 μg mL^−1^, 25 μg mL^−1^, 50 μg mL^−1^, 100 μg mL^−1^, 150 μg mL^−1^, whereas the medium without IO-CS nanoparticles was used for the control. 8 × 10^4^ ADSCs for MRI were incubated on 6-well plates, and 4 × 10^4^ ADSCs for Prussian blue staining were cultivated on 12-well plates. The labeled and unlabeled stem cells were trypsinized, and centrifuged, then dispersed in 0.5% agarose at 70 °C and gelated after cooling.

### 2.7 Prussian blue staining

Prussian blue staining was used to detect the intracellular IO-CS nanoparticles according to the ref. 26. 4 × 10^4^ cells labeled with 0 μg mL^−1^, 25 μg mL^−1^, 50 μg mL^−1^, 100 μg mL^−1^ IO-CS nanoparticles were fixed in 4% paraformaldehyde for 10 minutes and then washed 3 times with DD water following incubated with potassium ferrocyanide in hydrochloric acid for 20 min. The stained cells were rewashed 3 times with DD water and counterstained with nuclear fast red for 10 min. The labeled cells were examined with a light microscope to determine the intracellular iron oxide distribution.

### 2.8 Iron content in ADSCs

The cells were cocultured with IO-CS nanoparticles for 24 h, then washed with PBS 3 times and counted after trypsinization. The cells were resuspended in 0.5 mL of *aqua regia* and digested at room temperature for 24 h. Then, the above solution was diluted to 5 mL, and the cell number was counted. The iron content was determined by ICP-MS, and the average iron content per cell was then calculated.

### 2.9 Viability

To determine cell viability, the ADSCs were seeded on 96-well plates with 5000 cells per well with various IO-CS nanoparticles concentrations. After incubation for 24 hours, the culture medium with iron contents of 0 μg mL^−1^, 25 μg mL^−1^, 50 μg mL^−1^, 100 μg mL^−1^, 150 μg mL^−1^, and 200 μg mL^−1^ was added to each well and incubated for 24 h. Later, 110 μL of CCK-8 (10% v/v) solution was added to each well and incubated for another 1.5 h. Then, 100 μL of the supernatant of each well was measured at 450 nm with a microplate reader. For the longitudinal measurement of cell viability on days 1, 3 and 6, cells were seeded on 12-well plates with 4 × 10^4^ cell per well, and the experimental groups were incubated with 50 μg mL^−1^ IO-CS nanoparticles 24 h after seeding.

### 2.10 Differentiation

Adipogenic, osteogenic and chondrogenic differentiation tests were performed to assess the effect of IO-CS nanoparticles on the differentiation potential of ADSCs. IO-CS nanoparticles-labeled ADSCs of 50 μg mL^−1^ and the control group were subjected to three types of induction mediums (adipogenic, osteogenic and chondrogenic medium). The ADSCs for adipogenic differentiation were seeded on 12-well plates with 4 × 10^4^ cells per well. Upon reaching 80% confluence, the culture medium was replaced with an adipose cell-induction culture medium containing 10% FBS, 100 U mL^−1^ penicillin/streptomycin (Sigma-Aldrich), 200 mM indomethacin (Sigma-Aldrich), 1 mM dexamethasone (Sigma-Aldrich), 0.5 mM 3-isobutyl-1-methylxanthine (Sigma-Aldrich), and 10 mg mL^−1^ insulin (Sigma-Aldrich) in DMEM. The cells were cultured for 2 weeks and then stained with Oil Red O. For osteogenic induction, the ADSCs were cultured in 6-well plates with 10^5^ cells per well. After reaching 80% confluence, the culture medium was replaced by bone cell-induction culture medium containing 10% FBS, 100 U mL^−1^ penicillin/streptomycin, 50 μg mL^−1^l-ascorbate (Aladdin), 0.1 μM dexamethasone, and 10 mM β-glycerophosphate in DMEM. The cells were cultured for 3 weeks. Alizarin red was used to stain the matrix mineralization of osteogenic differentiation. The ADSCs for chondrogenic differentiation were maintained in 6-well plates with 10^5^ cells per well. After reaching 60% confluence, the culture medium was replaced by a chondrocyte induction culture medium bought from Cyagen Biosciences Inc. The cells were cultured for 4 weeks and then stained with Alcian blue.

### 2.11 Surgical induction of the osteoarthritis (OA) animal model

All animal procedures were performed in accordance with the Guidelines for Care and Use of Laboratory Animals of Tongji University and approved by the Animal Ethics Committee of Tongji University and Shanghai East Hospital (Shanghai, China). The OA models were established in 10 week-old male rats with weights of 300–350 g (purchased from Shanghai Slack Experimental Animals Co., Ltd. and housed in groups of four rats per cage with access to a standard commercial diet and water *ad libitum*) through the surgical destabilization of the medial meniscus (DMM) according to the surgical procedure described previously.^34^ The rats were anesthetized by intraperitoneal injection of 1.5% pentobarbital sodium with 45 mg kg^−1^, and the right knee was shaved and disinfected in preparation for surgery. A longitudinal surgical incision of 1.5 cm was cut through the epidermis. The articular capsule immediately medial to the patellar ligament was incised to expose the joint cavity. After dissecting the fat pad, the medial meniscotibial ligament was transected with microsurgical scissors, and the medial meniscus was dislocated. The articular capsule and skin were closed with 6-0 suture. An alcohol cotton ball was used to clean up the anastomosis after surgery. Afterwards, the animals were allowed access to feed and water unrestrictedly.

### 2.12 *In vitro* and *in vivo* MRI imaging

For *in vitro* MRI study, the labeled doses of the IO-CS nanoparticles were 12.5, 25, 50, 100, and 150 μg mL^−1^ with 8 × 10^4^ cells on 6-well plates. The labeled and unlabeled stem cells were trypsinized, centrifuged, dispersed in 0.5% agarose at 70 °C and gelated after cooling. *In vitro* cellular imaging was operated on a 3 T MRI system using a longitudinal relaxation time (*T*_2_) weighted fast spin-echo sequence. The sequence parameters were as follows: TR = 4000 ms; TE = 67 ms; BW = 200 Hz per Pix, resolution = 0.2 mm × 0.2 mm × 1 mm. For *in vivo* MRI study, 2 × 10^6^ ADSCs labeled with 50 μg mL^−1^ IO-CS nanoparticles were obtained by trypsin and re-suspended in 100 μL of PBS. After 4 weeks, the OA rats were anesthetized with chloral hydrate solution with a content of 0.4 mg kg^−1^. Then, the labeled cell suspension was injected to an articular cavity with 27 gauge needles. The rats were imaged after anesthesia 1 day, 2 weeks, and 5 weeks post-injection. The MRI of the rat joints were obtained from a clinically used 3.0 T MRI imager (MAGNETOM Prisma, Siemens Healthcare, Erlangen, Germany) equipped with an animal coil. The *T*_2_-weighted TSE sequence parameters were listed as follows: TR = 4200 ms; TE = 75 ms; FOV = 140 mm × 105 mm; matrix size = 448 mm × 269 mm; slice thickness = 2.5 mm.

### 2.13 Histological assessment

At 8 weeks post-injection, the SD rats were killed by dislocating the cervical spine, and the distal femur and proximal tibia were then harvested. The tissues were washed three times with PBS to remove residual blood. The tissues were next fixed with 4% paraformaldehyde for three days and then transferred to the EDTA decalcification solution, which was renewed every four days until the needle could be loosely inserted into the bone. Then, the tissues were paraffin embedded and sliced, and the obtained sections were stained with Prussian blue and Safranin-O.

### 2.14 Statistical analysis

Data are presented as mean ± standard deviation (SD). Statistical analyses of data were performed using GraphPad Prism 5 (GraphPad Software Inc., San Diego CA). *p < 0.05 was considered statistically significant.

## 3. Results and discussion

**Fig. 1:**
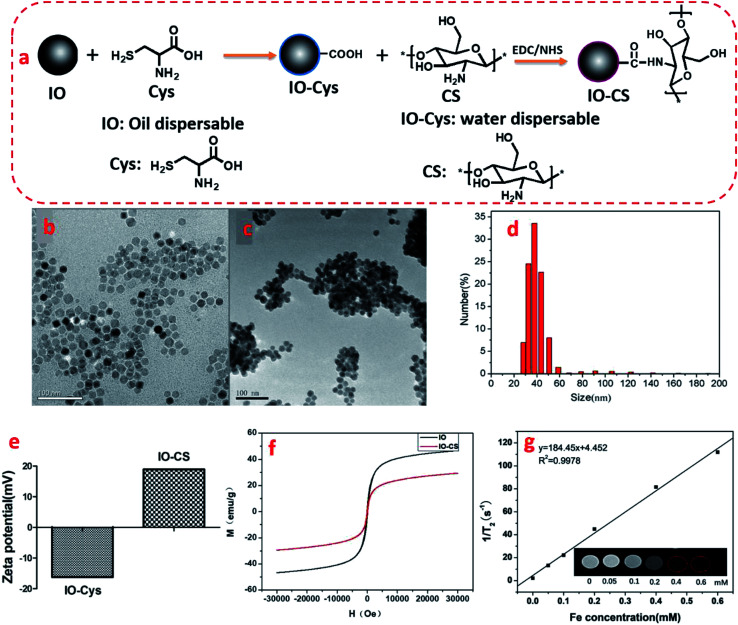
Schematic synthesis and characterization of IO and IO-CS nanoparticles. (a) Schematic synthesis of chitosan-modified iron oxide nanoparticles. TEM images of (b) IO nanoparticles and (c) IO-CS nanoparticles; (d) hydrodynamic diameter distribution of IO-CS nanoparticles by DLS. (e) Zeta potential of IO and IO-CS nanoparticles; (f) magnetization as a function of the applied magnetic field for IO and IO-CS nanoparticles; (g) MRI effect of IO-CS nanoparticles; the linear fitting of the 1/*T*_2_ as a function of Fe concentration. (The inset image was the *T*_2_-weight MRI analysis of IO-CS with various iron concentrations from 0–0.6 mM through a 0.5 T NMR analyzing and imaging system.)

The synthesis of IO-CS was demonstrated in Fig. 1a. To investigate the morphology of the nanoparticles, TEM was utilized to observe the IO and IO-CS nanoparticles, shown in Fig. 1b and c. IO nanoparticles were uniform with a size of 17 nm. After the simple modification of chitosan, the IO-CS nanoparticles showed similar uniformity with the same size. In addition, the mono-dispersion was confirmed by DLS tests (Fig. 1d). In Fig. 1e, the zeta potential results demonstrated a reversal from the negative charge of IO-Cys (−18 mV) to the positive charge of IO-CS nanoparticles (+12 mV). This was attributed to the negative –COOH groups of cysteine on the surface of the IO nanoparticles and the positive –NH_2_ groups of chitosan on the surface of the IO-CS nanoparticles, respectively. In conjunction with the FTIR spectrum (shown in Fig. S1†), it was confirmed that chitosan was successfully grafted onto the IO nanoparticles. The modification of IO nanoparticles with chitosan made the nanoparticle positively charged on the surface. The positive surface charge of the nanoparticles enhanced cellular uptake.^35,36^ To measure the magnetic property of IO and IO-CS nanoparticles, magnetization as a function of the applied magnetic field was obtained through measurement with a magnetometer (Fig. 1f). IO nanoparticles presented a saturation magnetization of 46.7 emu per g and for IO-CS nanoparticles was 29.4 emu per g. Although IO-CS nanoparticles showed a decrease in saturation magnetization, the nanoparticles could still be efficiently separated from the medium (data not shown). Furthermore, the MRI capacity of IO-CS nanoparticles was evaluated (Fig. 1g). The 1/*T*_2_ decreased linearly with increasing Fe concentration with an *R*_2_ of 184.45 mM^−1^ s^−1^, which was much higher than the commercial ferumoxytol (34.73 mM^−1^ s^−1^, shown in Fig. S2†). IO-CS nanoparticles demonstrated a clear hypointense MRI signal as the Fe concentration increased above 0.1 mM (inserted *T*_2_-weighted MR image in Fig. 1g). The corresponding *T*_2_-weighted MR images of 0.6 mM (33.6 μg mL^−1^) of Fe showed an obvious hypointense MRI signal. In summary, IO-CS nanoparticles were facilely prepared with good morphology, dispersity and imaging effect, which benefited clinical application.

**Fig. 2:**
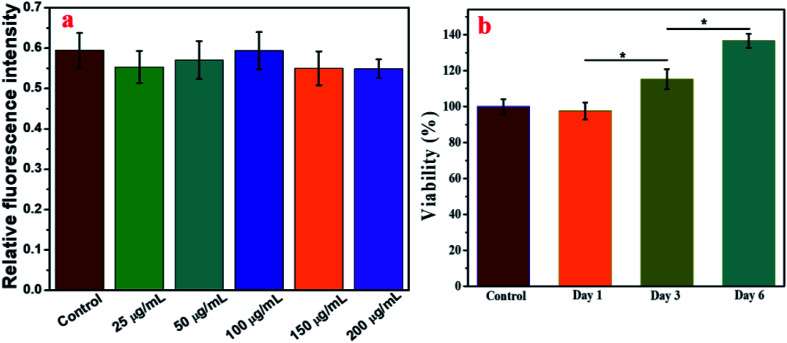
Cell viability of ADSCs after incubation with IO-CS nanoparticles. (a) Relative fluorescence intensity of ADSCs incubated with various concentrations of IO-CS nanoparticles for 24 h. (b) Proliferation profile of ADSCs incubated with 50 μg mL^−1^ on day 1, day 3 and day 6.

For clinical application, cell activity should receive much attention for the maintenance of cell function. Next, cell toxicity was evaluated for ADSCs with various feeding doses of 0 μg mL^−1^, 25 μg mL^−1^, 50 μg mL^−1^, 100 μg mL^−1^, 150 μg mL^−1^ and 200 μg mL^−1^. When the concentration of the IO-CS nanoparticles was no more than 200 μg mL^−1^, the cell activity of the ADSCs was comparable to the control group (Fig. 2a, no significant differences). There were no significant differences among the groups with various concentrations of IO-CS nanoparticles, which meant that incubation with IO-CS nanoparticles up to 200 μg mL^−1^ did not affect cell viability. This concentration range covered the required concentration of IO-CS nanoparticles for effective MR imaging (50 μg mL^−1^, shown in Fig. 4f and g). We also investigated the viability of ADSCs incubated with 50 μg mL^−1^ for 6 days. On day 1, the relative fluorescence intensity of the control group and the IO-CS nanoparticle group showed no significant difference. No obvious toxicity was observed. Later, ADSCs labeled with IO-CS nanoparticles exhibited very slow proliferation from day 3 and day 6.

**Fig. 3:**
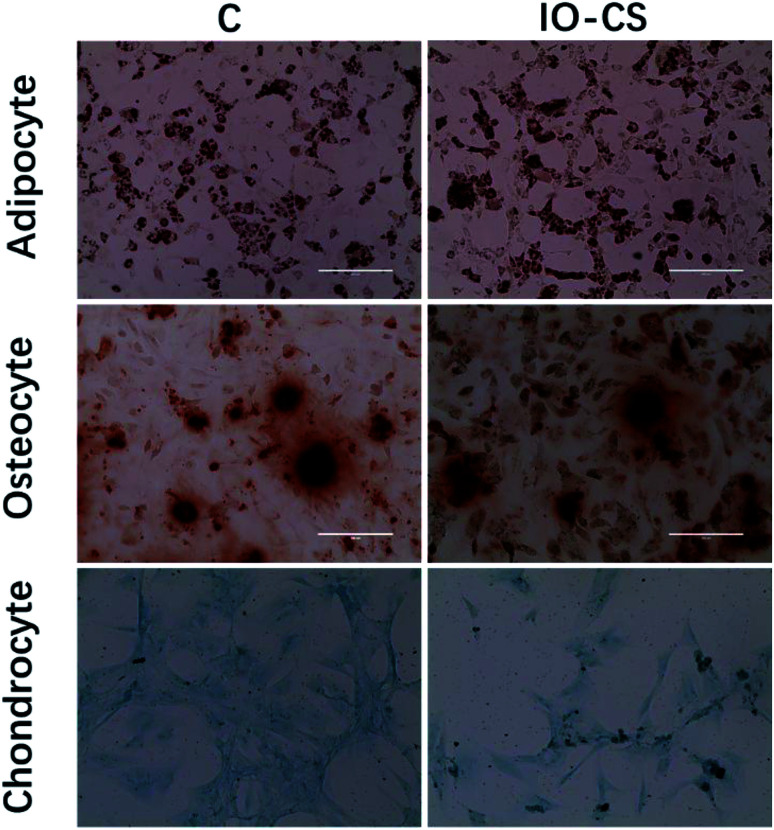
The multiple differentiation capacities of IO-CS nanoparticle-labeled ADSCs. ADSCs labeled with/without IO-CS nanoparticles were incubated with adipogenic, osteogenic and chondrogenic differentiation media for 3 weeks or 4 weeks. Oil Red O staining was for adipogenic differentiation, alizarin red staining for osteogenic differentiation and toluidine blue staining for chondrogenic differentiation, respectively.

For live stem cell imaging, it was essential for the stem cells to undergo differentiation and keep their functional capacity after labeling. To verify ADSCs stemness after labeling, adipogenic, osteogenic and chondrogenic differential medium was introduced to induce cell differentiation. IO-CS nanoparticle-labeled ADSCs demonstrated identical adipogenic differentiation capacity compared to unlabeled cells, which was demonstrated by Oil Red O staining with similar red colored lipid vacuoles (Fig. 3). In addition, there was no significant difference between the IO-CS nanoparticles labeled group and the control group (without labeled) from alizarin red staining and toluidine blue staining. These results demonstrated that IO-CS nanoparticles did not hinder the adipogenic, osteogenic or chondrogenic differentiation potential of ADSCs, which were consistent with other reports.^22^ Taking together, the viability test and multipotential differentiation measurement confirmed that IO-CS nanoparticles were safe and compatible vehicles for imaging.

**Fig. 4:**
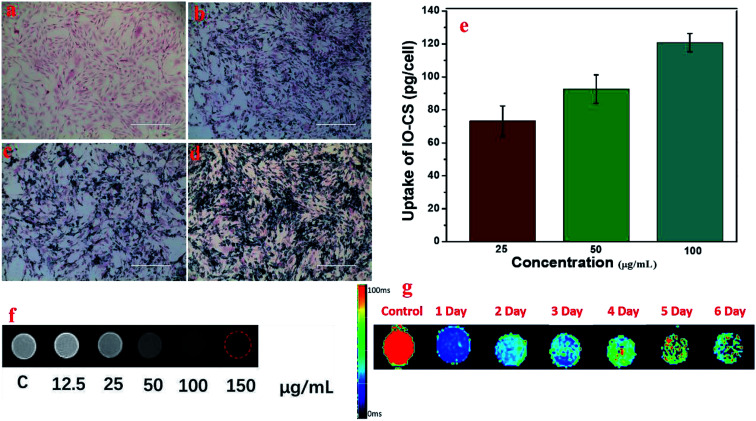
Labeling ADSCs with IO-CS nanoparticles for MRI. Prussian blue staining of ADSCs incubated with various concentrations of IO-CS nanoparticles, (a) 0 μg mL^−1^, (b) 25 μg mL^−1^, (c) 50 μg mL^−1^, (d) 100 μg mL^−1^. (e) Quantitative analysis of iron incorporation in ADSCs by ICP-MS. (f) The *T*_2_-weighted MRI of IO-CS-labeled ADSCs in a 3 T environment *in vitro*. 8 × 10^4^ ADSCs were treated with IO-CS nanoparticles at the iron concentrations of 12.5–150 μg mL^−1^ and ADSCs without labeling IO-CS nanoparticles were taken as the control. (g) The MRI transverse *R*_2_ maps to visualize ADSCs with and without labeling. Scale bar: 400 μm.

To visualize the magnetic nanoparticles in cells, Prussian blue staining was utilized to detect the internalization of iron oxide nanoparticles in ADSCs *in vitro*. Optical microscope images demonstrated the status of ADSCs post incubation with various feeding doses, shown in Fig. 4a. ADSCs demonstrated good morphology after carrying IO-CS nanoparticles, and the dark blue crystals were clear evidence of the internalization of IO-CS nanoparticles. Almost all the cells were efficiently labeled with IO-CS nanoparticles even for the lowest labeling dose (25 μg mL^−1^). In addition, it revealed that the staining color was darker when cells were fed with a higher IO-CS concentration, which meant more uptake of Fe. The morphology of the cells did not change when fed more magnetic nanoparticles. It demonstrated that the chitosan-modified IO-CS nanoparticles enhanced the uptake of iron nanoparticles significantly when compared to commercial ferumoxytol (shown in Fig. S3†). Quantitative evaluation was performed by detection with ICP-MS tests. As expected, the iron load of IO-CS nanoparticles was augmented from 73 pg per cell to 121 pg per cell when the cells were fed with increasing Fe doses from 25 μg mL^−1^ to 100 μg mL^−1^. Our chitosan-modified IO-CS nanoparticles showed much higher cell uptake of nanoparticles than previous reports, with less than 20 pg per cell at the same dose of 25 μg mL^−1^.^22,25,26^ Later, IO-CS nanoparticle-labeled ADSCs were imaged with MRI. The cell samples fed with more than 12.5 μg mL^−1^ Fe demonstrated the detectable signal intensity. The hypointensity MRI signal of localized regions increased as the feeding amount increased (Fig. 4f). It showed distinct hypointense signals at feeding doses of more than 25 μg mL^−1^. Next, the duration that cells could be detected by MRI was measured. *R*_2_ maps showed that the labeled ADSCs were detectable by MRI during the 6 days, as shown in Fig. 4g. Considering the imaging effectiveness and cell biocompatibility, the concentration of 50 μg mL^−1^ was fixed for further biological evaluation.

**Fig. 5:**
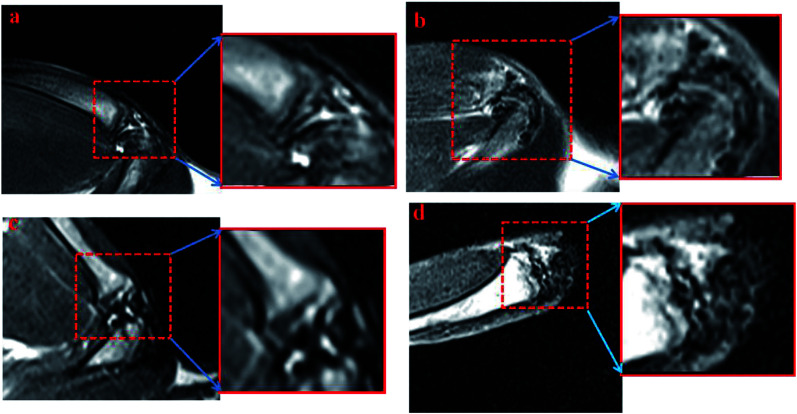
MRI of rat cartilages that were injected with IO-CS-labeled ADSCs *in vivo*. 10 week-old SD rats suffering from post-traumatic osteoarthritis were injected with PBS (control group) and 2 × 10^6^ ADSCs in right legs (ADSCs labeled with 50 μg mL^−1^ IO-CS, experimental group). MRI was examined at 24 h, 2 weeks and 5 weeks. (a) Control; (b) 24 h; (c) 2 weeks; (d) 5 weeks.

Later, the IO-CS signal was tracked by MRI *in vivo*. On the first day of injection with labeled ADSCs, it showed a local hypointensity signal in the articular cavity (the control group did not show any MRI signal). IO-CS nanoparticle-labeled ADSCs injected in osteoarthritic cartilage were distributed in the intra-articular area and could be clearly distinguished from surrounding tissues. After 2 weeks of injection, the hypointensity signal was still distinct from around tissues with local enrichment. Most of the MRI signal came from the joint cavity. The hypointensity signal was still detectable after 5 weeks of labeling, although the signal was dispersive compared to in the beginning. It could be noted that the navigation of IO-CS nanoparticles and metabolic consumption of Fe caused the decrease of signal intensity. The results were consistent with *in vitro* data, and histologic analysis confirmed the spread of IO-CS nanoparticles in the whole joint. For the long recovery time of renewing a cartilage tissue, it is essential to keep a stable imaging signal. Therefore, our IO-CS nanoparticles could serve as an effective and long-term MRI agent of ADSCs for cartilage regeneration (Fig. 5).

**Fig. 6:**
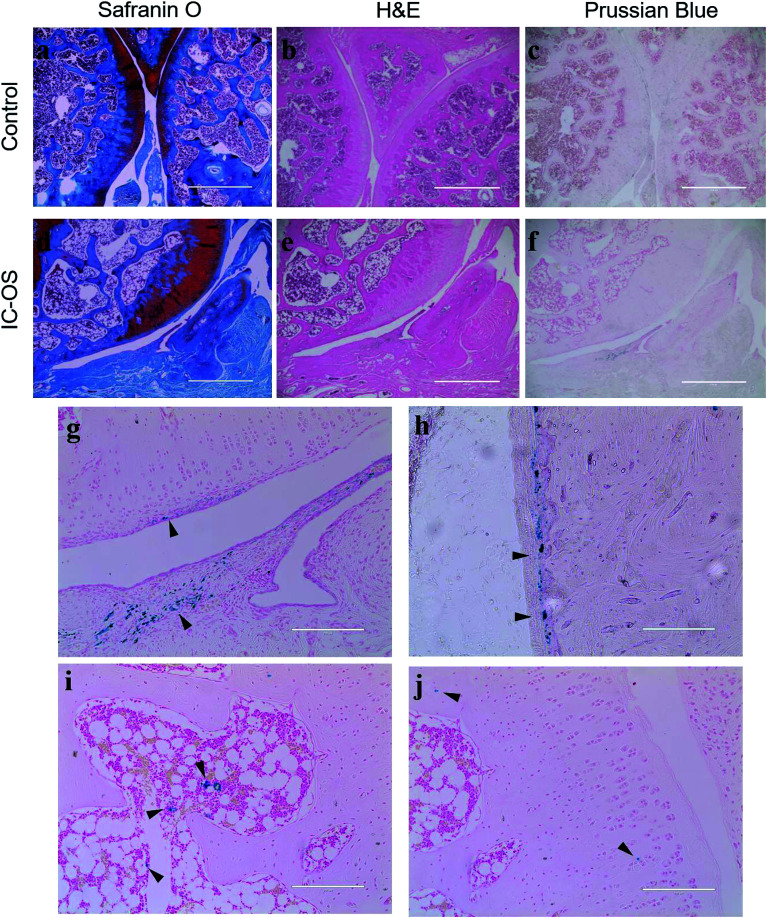
Histological staining of SD rat cartilage after 8 weeks post-injection. Representative Safranin-O staining, HE staining and Prussian blue staining of the control group (a–c) and experimental group (d–f). The positive Prussian blue staining results of the experimental group were shown in the high magnification field (g–j) and marked with arrows. Scale bar: (a–f) 100 μm, (g–j) 200 μm.

To determine the distribution of the IO-CS nanoparticles in the joint accurately, articular cartilage samples were harvested for H&E and Safranin-O staining. In the control group, the normal articular cartilage was intact and the surface was continuous; Safranin-O stain depletion displayed the front of the tibial plateau (Fig. 6a and b). In the experimental group, the cartilage layer was thicker compared to the normal group, with a layer of regenerated fibrous cartilage on the surface, and the original part of the dislocated meniscus was filled with synovium (Fig. 6d and e). Prussian blue was carried to verify the biodistribution of IO-CS nanoparticles. At low magnification (4 × 10), positive cells were observed in the cartilage superficial layer and the synovial sublining layer in the experimental group (Fig. 6f and g), which was consistent with results reported previously.^37^ No positive staining was found in the control group (Fig. 6c). At high magnification (20 × 10), we found the positive staining of enrichment in the periosteum. Murphy *et al.* reported this phenomenon on short-term tracking in the caprine model,^38^ while it was verified in our long-term tracking results. This was also consistent with previous MRI results, which may suggest that the periosteum plays an important role in both early and late post-traumatic events. T. Maerz *et al.* reported that the myotendinous junction (MTJ) of the quadriceps was also an important target in the acute mobilization of stem cells after anterior cruciate ligament transection (ACLT).^39^ Unfortunately, no significant positive staining was found in the muscle or tendon, which was probably due to the difference in surgery and time. Surprisingly, abundant positive staining was found in part of the bone marrow cavity (Fig. 6i), which we considered to be of great significance after excluding non-specific positive staining. Many studies had reported that bone marrow lesions (BMLs) played an important role in the symptoms of OA.^40–42^ Campbell *et al.* reported the alterations of bone marrow mesenchymal stem cells at the site of the bone marrow regions.^43^ However, no obvious BML was observed in this experiment, so further study was still needed to explore whether the injected allogenic stem cells could prevent or repair BMLs. In addition, sporadic positive staining was found in the bone matrix and the cartilage extracellular matrix. Although there has been literature showing that there are small tiny channels in the subchondral bone and cartilage that allowed for the exchange of small molecules,^44,45^ this was still too narrow for the cells. Another explanation is that osteocytes could move through the lacunar-canalicular system.^46^ Due to the low number of positive staining, we think the significance of this phenomenon is not as large as expected, and the movement of iron nanoparticles released through extracellular vesicles or engulfed by macrophages needs to be discussed. In any case, these results suggest that OA is a total joint disease rather than simple cartilage wear.^47^ In general, our study provided a novel method for the intra-articular injection tracking of stem cells, which is long-term and stable without affecting the stemness of exogenous stem cells. However, it is challenging to continuously track the fate of cells in both temporality and space, and some important signals may be missed due to the restriction of biopsy and MRI resolution. MRI requires more precision to help judge or replace it with high-precision CT. In addition, some distal organs need to be evaluated. It should be noted that whether the ADSCs were alive upon intra-articular injection was difficult to conclude. A limitation of this study is that the IO-CS-labeled ADSCs negotiate with IO-CS all the time. IO-CS released from dead ADSCs could be taken up by other cells or spread in the cartilage matrix. Other labeling approaches such as luciferase-labeled ADSCs could be utilized to monitor the cell survival *in vivo*. Even so, this is still a novel labeling method, which can provide much support for the study of the cell therapy of OA.

## 4. Conclusion

In summary, we developed a good MRI reagent with ease of availability, good biocompatibility and efficient labeling capacity, aimed at enhancing cell uptake and retention to prolong the cell tracking time. The chitosan-modified IO-CS nanoparticles demonstrated great morphology and dispersibility, with long-lasting MR imaging effect *in vitro* and *in vivo*. ADSCs showed the efficient uptake of IO-CS nanoparticles and good viability after labeling. Furthermore, ADSCs labeled with IO-CS nanoparticles showed multipotency without affecting stemness, including adipogenic, osteogenic and chondrogenic differentiation. Also, MRI was used to track IO-CS nanoparticle-labeled ADSCs for 6 days *in vitro* and for 5 weeks post intra-articular injection *in vivo*. It was confirmed that IO-CS nanoparticles spread in the joint capsule, including the cartilage superficial layer, synovial sublining layer, periosteum and bone marrow cavity. This study provided an efficient and stable tracer for ADSCs *in vitro* and *in vivo* after intra-articular injection in the OA rat model. It demonstrated the potential applications of IO-CS nanoparticles in labeling and tracking ADSCs in the articular capsule for pre-clinical and clinical studies.

## Conflicts of interest

There are no conflicts to declare.

## Supplementary Material

RA-009-C8RA09570A-s001
