# Supplementary material for: Intra-articular tracking of adipose-derived stem cells by chitosan-conjugated iron oxide nanoparticles in a rat osteoarthritis model
Source: RSC Adv. 2019 Apr 16;9(21):12010–9. doi: 10.1039/c8ra09570a (PMC9063530; doi:10.1039/c8ra09570a)
Supplement: RA-009-C8RA09570A-s001 [file RA-009-C8RA09570A-s001.pdf]

Electronic Supplementary Material (ESI) for RSC Advances.
This journal is © The Royal Society of Chemistry 2019

Intra-articular tracking of adipose-derived stem cells by chitosan conjugated iron oxide nanoparticles in a rat osteoarthritis model

Meihua Xie,‡a ShuLin Luo,‡b Ying Li,‡b Laiya Lu,[b] Cuijun Deng,[a] Yu Cheng*[a] and Feng Yin*[b]

[a] Shanghai East Hospital; The Institute for Biomedical Engineering & Nano Science, Tongji University School of Medicine, Shanghai, 200120, China; [b] Department of Joint Surgery, Shanghai East Hospital, School of Medicine, Tongji University, Shanghai, China; Corresponding authors: 001yinfeng@sina.com; yucheng@tongji.edu.cn

‡ The authors contributed equally to this work.

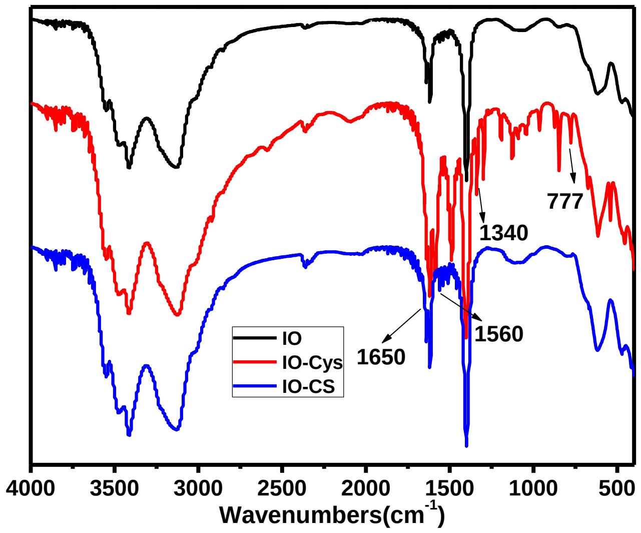

Figure S1. FTIR spectrum of IO, IO-Cys and IO-CS. The peak appeared at 777 cm^{-1} was due to stretch of C–S after conjugation of cysteine on the surface of IO. -COOH group of cysteine appeared at 1340 cm^{-1}[1]. C=O in amide group (amide I band) appeared at 1650 cm^{-1}after modification of IO with chitosan. The peak at 1560 cm^{-1} was attributed to NH-bending vibration in amide group[2].

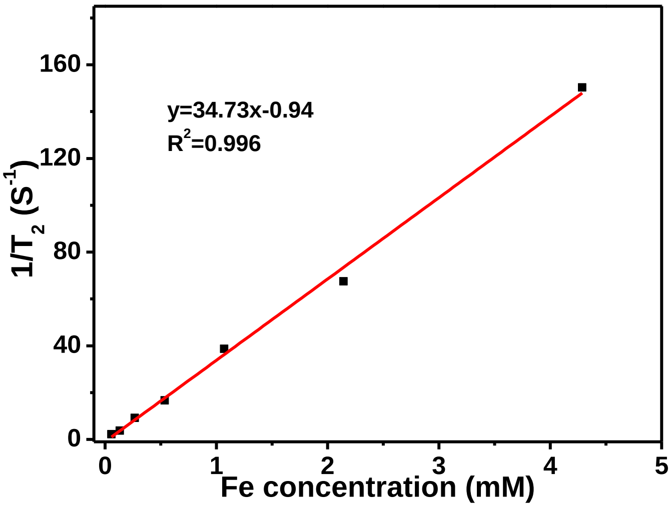

Figure S2. The linear fitting of 1/T2 as a function of Fe concentration from 0-0.6 mM at 0.5 T NMR analyzing and imaging system.

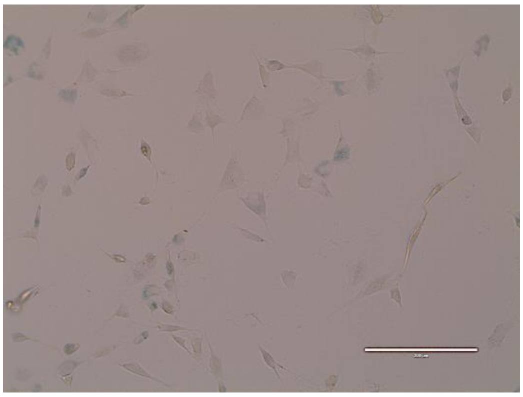

Figure S3. Prussian blue staining of ADSCs incubated with 50 μg/mL ferumoxytol.

Reference

[1] J. G. Parsons, K. M. Dokken, J. Mcclure, J. L. Gardea-Torresdey. *Polyhedron*. 2013;**56**:237-42.

[2] A. Pawlak, M. Mucha, *Thermochimica Acta*. 2003;**396**:153-66.
